# Foscarnet eyedrops for the treatment of refractory herpetic keratitis

**DOI:** 10.1186/s12348-024-00395-0

**Published:** 2024-10-23

**Authors:** Caroline C. Awh, Austen N. Knapp, Jeffrey M. Goshe, Craig W. See, Careen Y. Lowder

**Affiliations:** Cole Eye Institute, Cleveland Clinic, i-32, 9500, Euclid Ave, Cleveland, OH 44195 USA

## Abstract

**Purpose:**

The purpose of this case series is to describe the clinical course of patients receiving foscarnet eyedrops for the treatment of refractory herpetic keratitis.

**Observations:**

Six patients diagnosed with herpetic keratitis were treated with foscarnet 24 mg/mL (2.4%) eyedrops with resulting improvement in keratitis.

**Conclusion:**

Topical foscarnet may be a safe and effective treatment for herpetic keratitis in conjunction with, or as an alternative to, conventional antiviral therapy. This is an off-label use of foscarnet.

## Introduction

The first-line treatment for keratitis due to herpes simplex virus (HSV) or varicella zoster virus (VZV) is systemic antiviral therapy with acyclovir or its related drugs [1]. However, resistance to these antivirals is increasingly recognized, in part due to the long-term use of antiviral therapy for HSV and VZV prophylaxis [2]. Adjunctive and alternative therapy is necessary in order to manage patients with infections nonresponsive to conventional therapy. In this report, we describe the clinical course of 6 patients with refractory herpetic keratitis who had a favorable clinical response to foscarnet eyedrops.

## Methods

The Cleveland Clinic Institutional Review Board approved this retrospective interventional case series and deemed that informed consent was not required for this study. The study was performed in compliance with HIPAA guidelines and with the tenets of Declaration of Helsinki. In this case series, we reviewed the charts of patients who presented to the Cleveland Clinic Cole Eye Institute, were diagnosed with herpetic keratitis, and received treatment with topical foscarnet eyedrops. Six patients were identified. The following data was collected: age, sex, presentation, prior treatments, medical interventions, clinical course, and duration of treatment and follow-up.

## Results

6 patients (3 males and 3 females, with an average age of 73 [range: 48–89]) were included in the study. Patient clinical characteristics are summarized in Table 1. All patients presented with persistent keratitis (3 with recurrent/persistent pseudodendritic lesions, 1 with a persistent geographic defect, 2 with corneal edema and endotheliitis) despite treatment with therapeutic doses of oral guanosine analogues (4 on valacyclovir 1 g TID, 1 on acyclovir 800 mg QID, 1 on famciclovir 500 mg TID). Only 1 patient presented on topical antivirals (ganciclovir 0.15% gel 5x/day). One patient had a history of ganciclovir 0.15% gel and trifluridine eyedrop use but was not on any eyedrops at the time of presentation. Three patients had HSV-positive cultures from ocular specimens (2 from aqueous samples, 1 from corneal scraping). Three patients had presumed VZV keratitis with pseudodendritic lesions, without VZV-positive ocular cultures.

**Table 1:** Clinical characteristics of patients treated with foscarnet eyedrops

Pt. No., Age (Y), sex	Diagnosis and method of diagnosis	Exam findings	Prior treatment	Treatment course	Outcome after foscarnet eyedrops	Follow-up course
1. 85 M	Recurrent HSV keratitis and scleritis, HSV + epithelial culture	Non-improving geographic defect with persistent scleritis	Valacyclovir PO 1 g TID x 10 days, increased to valacyclovir PO 2 g TID x 1 month, switched to famciclovir PO 500 mg TID x 2 weeks; allergic to ganciclovir gtts	Underwent amniotic membrane graft and initiated foscarnet drops q2hr with taper to QID	Epithelium closed in 2 weeks	Discontinued foscarnet gtts after 4 months due to logistical constraints; s/p permanent tarsorrhaphy and is maintained on famciclovir PO 500 mg daily
2. 67 F	Presumed VZV	Persistent pseudodendrites	Acyclovir PO 800 mg QID	Started foscarnet gtts q2hrs and switched acyclovir to valacyclovir 1 g BID	Resolution of lesions in 2.5 weeks, discontinued foscarnet gtts	Maintained on acyclovir 800 mg BID x 2 months, followed by new pseudodendritic lesion. Restarted foscarnet gtts but developed irritation; switched to ganciclovir gtts 5x/day. Currently on ganciclovir gtts 5x/day and valacyclovir PO 1 g TID
3. 69 M	Recurrent HSV endotheliitis, HSV + aqueous sample	Corneal edema and haze	Valacyclovir PO 1 g TID x 2 months	Started foscarnet gtts QID and switched to PO famciclovir 500 mg TID	Resolution of edema and haze in 5 months	Maintained on foscarnet gtts BID and famciclovir PO 500 mg daily
4. 89 F	Presumed VZV keratitis	Recurrent pseudodendrites	Valacyclovir PO 500 mg TID, ganciclovir gtts 5x/day x 4 months, history of repeat epithelial debridements	Started foscarnet gtts q2hr, stopped ganciclovir gtts, continued famciclovir PO 500 mg TID	Slow resolution of pseudodendrites in 1 year (had one recurrence when patient stopped foscarnet gtts, which resolved once foscarnet gtts were restarted)	Developed calcific band keratopathy 17 months after initiation of foscarnet gtts; discontinued foscarnet gtts and underwent debridement; maintained on famciclovir 500 mg daily
5. 79 M	Presumed VZV keratitis	Worsening pseudodendrite (acute onset after phototherapeutic keratectomy for anterior stromal scar)	Valacyclovir PO 1 g TID x 6 weeks	Started foscarnet gtts q2hr tapered to QID, continued valacyclovir 1 g TID	Resolution of pseudodendrite in 2.5 months; ran out of foscarnet gtts and remained off drops without recurrence	Maintained on valacyclovir 1 g daily
6. 48 F	HSV, HSV + aqueous sample	Corneal edema and haze	Valacyclovir PO 1 g TID	Started foscarnet gtts QID and tapered Valtrex to 500 mg TID	Resolution of corneal haze in 2 months	Maintained on foscarnet gtts TID and valacyclovir 500 mg daily

*Abbreviations* HSV, herpes simplex virus; VZV, varicella zoster virus; PO, oral; gtts, drops

**Fig. 1:**
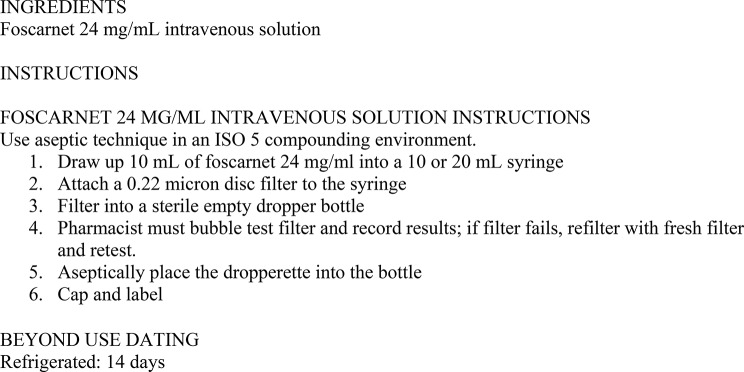
Instructions for Foscarnet 24 mg/mL Topical Ophthalmic Drops

All 6 patients were started on foscarnet 24 mg/ml eyedrops, formulated by the Cleveland Clinic pharmacy following a protocol detailed in Fig. 1. The drops are compounded by filtering Foscavir (foscarnet sodium 24 mg/mL manufactured by Hospira, Inc.) into a sterile empty dropper bottle made of zinc stearate-free low-density polyethelyene. The pH of the formulated drops is 7.4 with an osmolarity of 319.89, and the drops have a recommend beyond use date of 14 days under refrigeration. Patients were started on foscarnet eyedrops at a frequency ranging from 4x/day to every 2 h. At the time of initiation of foscarnet eyedrops, 3 patients had additional therapeutic interventions (1 underwent amniotic membrane graft, 1 switched from oral acyclovir 800 mg QID to oral valacyclovir 1 g BID, 1 switched from oral valacyclovir 1 g TID to famciclovir 500 mg TID).

**Fig. 2:**
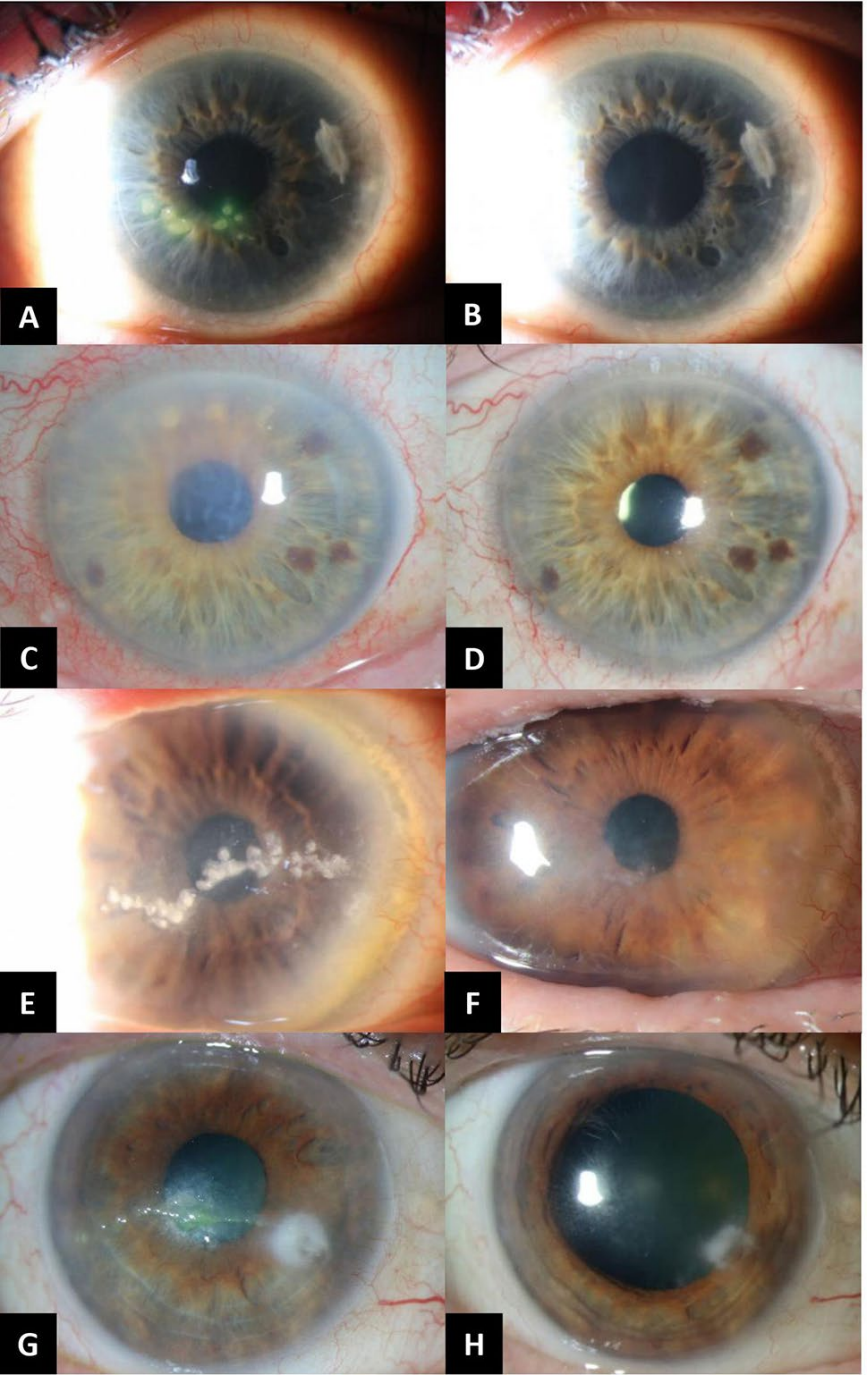
Patient 2 before (A) and after (B) 2 months of foscarnet drops, demonstrating resolution of pseudodendritic lesions. Patient 3 before (C) and after (D) 5 months of foscarnet drops, demonstrating resolution of corneal edema and haze. Patient 4 before (E) and after (F) 17 months of foscarnet drops followed by debridement of calcific band keratopathy, demonstrating resolution of pseudodendritic lesions. Patient 5 before (G) and after (H) 2.5 months of foscarnet drops, demonstrating resolution of pseudodendritic lesions. No slit lamp photos were available for patient 1 and 6

All 6 patients had significant improvement of their clinical exam findings after the initiation of foscarnet eyedrops **(**Fig. 2**)**. At the time of their most recent follow-up visit, 2 patients have remained on topical foscarnet drops. The remaining 4 patients discontinued the drops due to irritation (1) and near or complete resolution of keratitis without recurrence (3). All 6 patients remain on an oral guanosine analogue at their final follow-up (3 on famciclovir 500 mg daily, 1 on valacyclovir 500 mg daily, 1 on valacyclovir 1 g daily, 1 on valacyclovir 1 g TID).

## Discussion

We report these cases to provide evidence for the safe and effective use of foscarnet eyedrops for herpetic keratitis refractory to conventional antiviral therapy. No randomized control trial has evaluated the use of foscarnet eyedrops, and we do not know the necessary duration of treatment or potential toxicity.

Systemic antiviral therapy is frequently utilized as a first-line treatment for herpetic keratitis. This is typically achieved with acyclovir, a guanosine analog, or its prodrug valacyclovir, followed by long-term lower-dose prophylactic treatment with the same antiviral [1, 2]. All 6 of our patients had persistent herpetic keratitis despite a therapeutic dose of oral guanosine analog therapy. Notably, none of our patients had a known immunosuppressive condition. Once thought to be a complication only for the immunocompromised, resistance to antiviral drugs is increasingly recognized in immunocompetent individuals with herpetic keratitis [2].

Multiple mechanisms play a role in antiviral drug resistance in herpetic keratitis. One mechanism is due to the cornea’s immune privilege status. In non-ocular herpetic infections, such as herpes labialis and genital herpes, viral replication is largely inhibited by local immune responses, and antivirals play more of a supportive role. In the cornea—an immunoprivileged site with reduced local immune response—viral control is much more dependent on antiviral sensitivity. However, prolonged use, such as long-term prophylactic use, can lead to resistant viral strains [3, 4].

At the enzymatic level, herpetic resistance to antiviral therapy is most commonly caused by reduced thymidine kinase (TK) activity [5]. Acyclovir is a guanosine analogue that requires phosphorylation by the viral thymidine kinase (TK) prior to conversion to its active form. The same is required of acyclovir’s prodrugs including valacylovir and famciclovir. Resistance to these medications can occur with viral mutations in TK. Foscarnet is a direct viral DNA polymerase inhibitor and does not require activation by TK. Thus, it may be an alternate antiviral therapy when resistance is due to a TK mutation [5, 6].

Foscarnet has low oral bioavailability and is most commonly administered intravenously. It has been reported to be a successful treatment for acyclovir-resistant herpetic keratitis [6]. However, systemic foscarnet is associated with significant systemic adverse effects including renal damage, nausea, and electrolyte derangements [7]. Additionally, it is administered intravenously three times daily, a significant burden to patients, who would require either admission to a hospital or a peripherally inserted central catheter (PICC) line. Five of our six patients tolerated topical foscarnet well without any reported adverse effect. One of our patients had resolution of presumed VZV pseudodendrites after 2.5 weeks of foscarnet eyedrops; however, the lesions recurred after discontinuation of the drops. Upon restarting the drops, the patient reported new irritation, and the drops were discontinued.

The safe and effective use of topical foscarnet for HSV-related corneal disease has been reported in the literature in a small group of case series and case reports. In 1992, Behrens-Bauman reported that topical foscarnet 3% was as effective as trifluridine 1% in the treatment of HSV dendritic keratitis, without any reported side effects [8]. In 1998, Fabricius studied varying concentrations of topical foscarnet in 10 patients with HSV keratitis and found that corneal toxicity developed in 3 of 6 patients using foscarnet 1.9% after 26–40 days of treatment, but the medication was well tolerated by all 7 patients using foscarnet 1.2% and 1.4% [9]. In 2001, Cao et al. found topical foscarnet 3% to be both safe and more effective than topical acyclovir 0.1% for HSV keratitis (65% cure rate versus 41.27% cure rate, respectively) [10]. In 2012, Yu et al. reported that while both topical foscarnet and topical ganciclovir led to significantly decreased corneal signs and symptoms of HSV epithelial keratitis, treatment with topical foscarnet led to significantly decreased recurrence rate [11]. Most recently, in 2022, De Clerck et al. published a case of a patient with genotypically proven acyclovir-resistant HSV keratitis successfully treated with topical foscarnet 1.2% five times daily [12].

Given the small number of patients in our group, the true incidence of toxicity to foscarnet 2.4% eyedrops remains unknown. While one of six patients (20%) is a relatively high percentage of adverse events, we are reassured that the one patient with toxicity had mild symptoms that resolved upon drug cessation. She complained of ocular irritation but did not present with any exam findings of allergic keratoconjunctivitis or corneal toxicity. We believe that the tolerance exhibited by our patients and those in prior reports is reassuring to continue investigating this treatment option for patients suffering from disease that is refractory to established treatments. Further studies investigating response at different dosages, such as the one conducted by Fabricius, should be conducted. As with any medication, all patients starting foscarnet eyedrops must be counseled on the risks and benefits of treatment.

The role of guanosine analogues and their derivatives are unclear when used in combination with foscarnet. All 6 of our patients are maintained on treatment regimens that include valacyclovir or famciclovir, despite the initial lack of response to these medications at therapeutic doses. A case from the dermatology literature in 1998 reported a patient with culture-proven acyclovir-resistant HSV-2 genital ulceration who was successfully treated by stopping oral acyclovir and starting topical foscarnet 2.4% cream. One month after treatment, sensitivity cultures revealed that the virus recovered sensitivity to acyclovir [13]. It is possible that in some of our patients, treatment of the resistant virus with the use of topical foscarnet allowed a guanosine analogue-sensitive strain to appear.

Another possible mechanism by which foscarnet eyedrops treat refractory herpetic disease is through synergy with other antivirals. All six of our patients were maintained on oral antiviral therapy with a guanosine analogue throughout their treatment course. In vitro and in vivo studies have shown guanosine analogues to be synergistic with other drugs that inhibit HSV and VZV replication through alternative mechanisms of action, including cidofovir, amenamevir, vidarabine, and docosanol [14–16]. Such studies have not been conducted on foscarnet, and understanding the interactions between foscarnet and the guanosine analogues would require extensive experimental investigations.

Patient 3 and 6 had HSV endotheliitis without epithelial disease, both of whom had resolution of their corneal haze after the addition of foscarnet eyedrops. Similarly, Fabricius treated 3 patients with disciform endotheliitis without epithelial disease with foscarnet 1.9% eyedrops, all of whom had resolution of their keratitis. Given that endotheliitis is due to active HSV infection in the anterior chamber [1], this suggest that foscarnet eyedrops may be able to penetrate the cornea and achieve adequate aqueous humor concentration. Topical antivirals vary in their ability to penetrate the cornea—while topical trifluridine can only achieve therapeutic concentrations in the anterior chamber when the cornea has been debrided or damaged, topical acyclovir has been shown to achieve therapeutic levels with an intact epithelium [17]. Ganciclovir 0.15% levels in aqueous humor have not been studied. Further studies are required to investigate the pharmacokinetics of foscarnet and its levels in aqueous humor following topical application.

A limitation of this case series is that no patient had pheno- or genotypically proven guanosine analogue resistance to HSV or VZV, and we assumed some level of resistance given the lack of response to therapeutic doses of oral antiviral medication. Of note, patient 3 and 6, both with persistent endotheliitis, were on prednisolone 1% drops in addition to oral antivirals at the time of initiation of foscarnet treatment. It is possible that in the absence of adequate antiviral penetration of the oral medications, the virus was able to continue proliferation in the anterior chamber in part due to this steroid use.

Another limitation is that prior to the initiation of foscarnet eyedrops, only 1 of the 3 patients with presumed VZV pseudodendrites had been trialed on topical ganciclovir, which has been reported to be an effective treatment for VZV pseudodendrites that are resistant to other oral or topical antivirals [17]. As a commercially available product in the United States, ganciclovir 0.15% ophthalmic gel may be a more accessible treatment option for patients with refractory herpetic keratitis.

Another limitation is that our 3 patients with VZV pseudodendrites did not have positive VZV ocular cultures, and thus the diagnosis of VZV was presumed. However, patient 2 had a remote case of VZV affecting V2 on the ipsilateral side, and patient 4 and 5—who did not have any known VZV history—both had classic VZV pseudodendritic features and responded to antiviral therapy. These cases suggest that a classic pseudodendritic lesion should raise suspicion for occult VZV, even in the absence of a clinical history of shingles.

In conclusion, supplemental treatment with foscarnet eyedrops led to the near or complete resolution of active keratitis in all 6 of our patients who had previously been resistant to other antiviral medications. This is an off-label use of foscarnet. We hope that the favorable outcomes of our patients lead to increased awareness of an additional treatment option for ophthalmologists when faced with managing these difficult cases.

## Data Availability

Data sharing is not applicable to this article as no datasets were generated or analyzed during the current study.
